# Supplementary material for: Identification of photoperiod-induced specific miRNAs in the adrenal glands of Sunite sheep (Ovis aries)
Source: Front Vet Sci. 2022 Jul 22;9:888207. doi: 10.3389/fvets.2022.888207 (PMC9354845; doi:10.3389/fvets.2022.888207)
Supplement: Supplementary file 1 [file Data_Sheet_1.ZIP › Supplementary file/miRDeep2 file.zip!/miRDeep2 file/Know_Structure/SP42A1/oar-mir-380.pdf]

miRBase precursor : oar-mir-380
Total read count : 2091
oar-miR-380-5p read count : 20
oar-miR-380-3p read count : 2070
remaining reads : 1

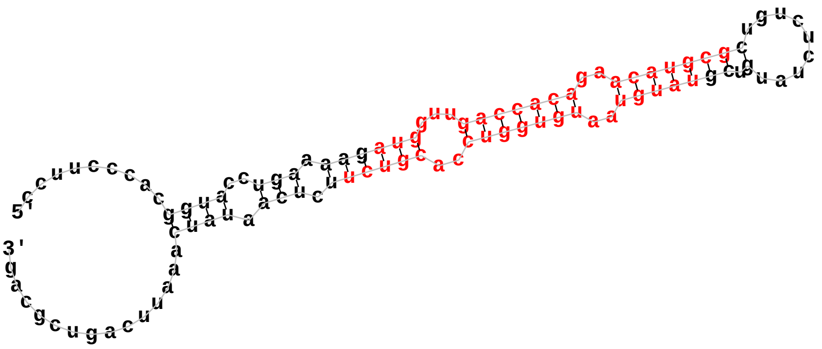

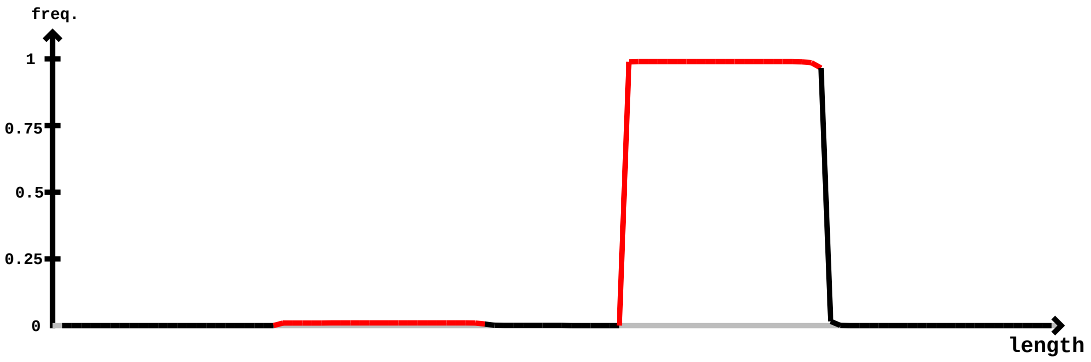

oar-miR-380-5p

oar-miR-380-3p

```
5'- ccuucccacggguaccugaaaagaugguugaccacagaacaugcgcugucucuaugucguauguaaugugguccacgucuucucaauaucaaauucagucgcag   -3'    exp
    .........((((..(((.((((((...(((((((..((((((((.........).)))))))..)))))))..)))))).))).))))..............    reads     mm     sample
    ......................augguugaccacagaacaug.............................................................    1         0      seq
    ......................augguugaccacagaacaugc............................................................    8         0      seq
    ......................augguugaccacagaacaugcg...........................................................    10        0      seq
    ......................augguugaccacagaacaugcgc..........................................................    1         0      seq
    ...........................ugaccacagaacaugcgcugucucu...................................................    1         0      seq
    ..........................................................uauguaaugugguccacg...........................    2         0      seq
    ..........................................................uauguaaugugguccacgu..........................    7         0      seq
    ..........................................................uauguaaugugguccacguc.........................    42        0      seq
    ..........................................................uauguaaugugguccacgucu........................    1984      0      seq
    ..........................................................uauguaaugugguccacgucuu.......................    32        0      seq
    ..........................................................uauguaaugugguccacgucuuc......................    1         0      seq
    ...........................................................auguaaugugguccacgucu........................    2         0      seq
```
